# Immunologic characteristics of HIV‐infected individuals who make broadly neutralizing antibodies

**DOI:** 10.1111/imr.12504

**Published:** 2017-01-30

**Authors:** Persephone Borrow, M. Anthony Moody

**Affiliations:** ^1^ Nuffield Department of Clinical MedicineUniversity of OxfordOxfordUK; ^2^ Duke University Human Vaccine Institute and Departments of Pediatrics and ImmunologyDuke University School of MedicineDurhamNCUSA

**Keywords:** autoantibody, broadly neutralizing antibody, CD4^+^ T follicular helper cell, CD4^+^ T follicular regulatory cell, human immunodeficiency virus, regulatory T cell

## Abstract

Induction of broadly neutralizing antibodies (bnAbs) capable of inhibiting infection with diverse variants of human immunodeficiency virus type 1 (HIV‐1) is a key, as‐yet‐unachieved goal of prophylactic HIV‐1 vaccine strategies. However, some HIV‐infected individuals develop bnAbs after approximately 2‐4 years of infection, enabling analysis of features of these antibodies and the immunological environment that enables their induction. Distinct subsets of CD4^+^ T cells play opposing roles in the regulation of humoral responses: T follicular helper (Tfh) cells support germinal center formation and provide help for affinity maturation and the development of memory B cells and plasma cells, while regulatory CD4^+^ (Treg) cells including T follicular regulatory (Tfr) cells inhibit the germinal center reaction to limit autoantibody production. BnAbs exhibit high somatic mutation frequencies, long third heavy‐chain complementarity determining regions, and/or autoreactivity, suggesting that bnAb generation is likely to be highly dependent on the activity of CD4^+^ Tfh cells, and may be constrained by host tolerance controls. This review discusses what is known about the immunological environment during HIV‐1 infection, in particular alterations in CD4^+^ Tfh, Treg, and Tfr populations and autoantibody generation, and how this is related to bnAb development, and considers the implications for HIV‐1 vaccine design.

This article is part of a series of reviews covering B cells and Immunity to HIV appearing in Volume 275 of *Immunological Reviews*.

## 1 Introduction

Human immunodeficiency virus type 1 (HIV‐1) infection remains a persistent global health threat, and approximately two million new adult infections occur each year.^1^ Education, identification and treatment of infected persons with antiretroviral drugs, male circumcision, condoms, and needle exchange programs have been effective at curtailing the epidemic, but declines in the rate of new infections have plateaued, and it appears unlikely that the goal of <500 000 new adult infections per year will be achieved by 2020. An effective HIV‐1 vaccine could contribute to further reductions of infections as part of a coordinated prevention strategy,^2^ but to date, testing of candidate vaccines in efficacy trials has been disappointing 3, 4, 5, 6, 7 with only one trial showing any degree of vaccine efficacy.^8^

A number of antibody‐mediated functions against HIV‐1 have been studied, including virus capture and phagocytosis,^9^, ^10^ antibody‐dependent cell‐mediated virus inhibition,^11^, ^12^ antibody‐dependent cell‐mediated cytotoxicity (ADCC),^13^, ^14^ chemokine secretion of monocytes stimulated by antibodies,^15^ and virus neutralization.^16^, ^17^, ^18^ Neutralization has been shown to exert immune pressure on HIV‐1,^19^, ^20^ while the role of other antibody‐mediated functions in exerting immune pressure is unclear, primarily because antibodies that mediate ADCC and other activities often also neutralize.^21^ Regardless, multiple studies have demonstrated that virus neutralization is a driver of both virus and antibody diversity,^22^, ^23^, ^24^, ^25^, ^26^ although neutralization of autologous viruses does not always cause the extinction of susceptible virus populations in an infected individual.^25^

Because of their ability to neutralize many different circulating strains of HIV‐1, broadly neutralizing antibodies (bnAbs) are attractive targets for vaccine development.^27^ Passive infusion studies in animals have shown that bnAbs can prevent infection by intravenous^28^ and mucosal^28^, ^29^, ^30^, ^31^, ^32^, ^33^ challenge, suggesting that a vaccine that elicits robust and durable levels of bnAbs could be protective. Vaccine development strategies that leverage our understanding of antibody‐virus coevolution^34^ and that use knowledge of antibody‐antigen structure relationships^27^ are currently being tested in animal models^35^, ^36^ and human studies are planned. However, to date, no vaccine has reliably elicited bnAbs, and one possibility is that in addition to optimizing antigen structure, it may be necessary to recreate the immunological environment in which bnAbs develop. Understanding the conditions in which bnAbs have developed is a critical first step toward recreating those conditions.

## 2 Development of broadly neutralizing antibodies in HIV‐1 infected individuals

### 2.1 The antibody response in HIV‐1 infection

During acute HIV‐1 infection, there is a vigorous immune response that is unable to contain virus replication or the establishment of latency.^37^ In many cases, a single transmitted/founder virus establishes infection and then evolves within the host resulting in a diverse virus population.^38^ The earliest changes to the virus population are driven by the CD8^+^ T‐cell response that is induced as viremia increases^39^ and places strong selection pressure on the virus, resulting in complete turnover of the virus pool within the first few weeks of infection.^40^ The development of antibody responses follows a pattern, with antiviral antibodies being detected first as immune complexes^41^ followed by free antibody directed at the HIV‐1 envelope (Env) glycoprotein 41 (gp41) subunit,^41^, ^42^ and then by the development of Env glycoprotein 120 (gp120)‐binding antibodies.^41^ These early antibody responses do not neutralize or place selective pressure on virus evolution^41^; antibodies capable of neutralizing autologous viruses are not detectable until weeks to months after infection is established^19^, ^20^ and have little to no activity against heterologous HIV‐1 strains.^43^ The initial gp41‐directed antibody response is polyreactive and the antibodies are highly mutated,^42^ and evidence indicates that at least some early responding B cells are primed prior to infection by non‐HIV‐1 antigens such as proteins contained in intestinal microbiota.^44^

Acutely HIV‐1‐infected individuals do not make bnAbs,^41^, ^43^ but during chronic HIV‐1 infection, neutralization breadth develops to a greater or lesser degree in each person.^45^, ^46^, ^47^ Breadth of plasma neutralizing activity develops incrementally,^48^ and evidence suggests that protracted exposure to HIV‐1 Env is required.^22^, ^23^, ^24^, ^47^, ^48^, ^49^, ^50^ Neutralization breadth is not an all‐or‐nothing phenomenon—in one study, about half of a cohort of 205 chronically infected persons were capable of neutralizing about half of a panel of 219 hard‐to‐neutralize (tier 2) HIV‐1 isolates.^45^ When bnAbs arise, neutralization breadth is thought to be mediated by 1‐2 specificities per individual,^51^, ^52^ although serum mapping studies^53^, ^54^, ^55^, ^56^ and algorithms designed to deconvolute neutralization data^57^ suggest that in some individuals breadth may be mediated by three or more specificities of antibodies. In addition, the acquisition of additional neutralization breadth can continue during ongoing HIV‐1 infection. For example, in one infected individual (CH505), a CD4‐binding site‐directed bnAb developed^24^ that was aided in its evolution by a cooperating antibody lineage also directed at the CD4 binding site but that did not initially have neutralization breadth.^23^ During ongoing infection, this cooperating antibody lineage further evolved, developing bnAb activity.^22^ The latter example suggests that the development of neutralization breadth is a dynamic process and that conditions favorable to the evolution of bnAbs may persist during chronic infection in some individuals.

Despite the fact that B cells are not a primary target of HIV‐1 infection, virus replication and immune activation in HIV‐1 infection are associated with profound dysregulation of the B‐cell compartment. It is possible that trafficking of the negative regulatory factor (Nef) protein from HIV‐1‐infected macrophages to B cells may alter class switching and germinal center (GC) responses,^58^ but dysfunction is also likely driven by the early cytokine storm^59^ and ongoing immune dysfunction caused by HIV‐1 infection of T cells. B‐cell dysregulation is evidenced by the delayed antibody response in acute HIV‐1 infection,^41^ an increase in the proportion of activated memory B cells and exhausted B cells, non‐specific plasmablast activation (leading to polyclonal immunoglobulin production), and a decline in the frequency of long‐lived plasma cells.^42^, ^60^, ^61^, ^62^, ^63^ In addition, Env‐specific B cells are found in the activated and exhausted B‐cell subsets in viremic individuals.^64^ However, the B‐cell dysfunction in HIV‐1 infection does not prevent the generation of bnAbs, and the extent of dysregulation of circulating B‐cell subsets during chronic infection shows no correlation with neutralization breadth.^65^, ^66^ At this time, it is not known whether B‐cell dysfunction is necessary for the development of breadth in HIV‐1‐infected individuals.

### 2.2 Clinical characteristics of individuals making bnAbs

Longitudinal studies of HIV‐1 infection have allowed researchers to map the development of bnAbs.^22^, ^23^, ^24^, ^26^, ^48^ During acute/early HIV‐1 infection, high viral load and an early decline in circulating CD4^+^ T cells were associated with the development of neutralization breadth,^48^, ^50^ whereas individuals who had low or undetectable viral loads, such as long‐term non‐progressors, were found to have less neutralization breadth.^49^ Time since infection also correlated with the development of breadth,^56^ although whether this was due to the need for protracted Env exposure, persistent immune perturbation caused by HIV‐1 infection, or both is not clear.

The development of neutralization breadth does not appear to impact the progression of HIV‐1 disease^48^, ^67^; for example, many individuals in these longitudinal cohorts went on antiretroviral therapy as part of standard of care during the course of study,^48^, ^50^ including those with high degrees of breadth.^48^ Examination of viruses and antibodies taken from the same HIV‐1‐infected individuals has consistently shown that circulating bnAbs cause extinction of susceptible virus populations despite ongoing viremia,^23^, ^24^, ^25^ although viruses susceptible to less potent neutralizing antibodies continue to circulate.^25^ Whether individuals who have developed bnAbs exhibit a reduced susceptibility to HIV‐1 super‐infection has not been addressed, although as bnAbs constitute only part of the overall HIV‐specific antibody response they may not reach sufficient titers to do so. The lack of clinical impact of bnAbs is consistent with early bnAb infusion studies in animal models^68^ and humans^69^ that did not show a lasting impact on HIV‐1 viral loads, although newer, more potent bnAbs appear to have a more persistent impact on viral load^70^, ^71^, ^72^ and subsequent antibody development.^73^ At this time, ongoing clinical trials are being conducted to determine if bnAb infusion can augment other anti‐HIV‐1 therapies.

The duration and magnitude of Env exposure that is permissive for bnAb development is not completely known. Longitudinal studies suggest that bnAbs do not develop until at least 2 years after HIV‐1 infection, but a study of two HIV‐1‐infected individuals who made Env gp41 membrane proximal external region (MPER) bnAbs showed that activity developed at about 1 year of infection while they were not on antiretroviral therapy.^26^ A second study compared individuals taking and not taking antiretroviral therapy and determined that the frequency of individuals making bnAbs was similar between the groups,^74^ although not all individuals taking antiretrovirals were completely suppressed. Thus, while prolonged Env exposure appears to be highly correlated with the development of neutralization breadth, there does appear to be a pathway to bnAb development in some individuals that lack prolonged or high‐level Env exposure.

Common demographic characteristics do not appear to influence bnAb development. In a large longitudinal study of African HIV‐1‐infected individuals, there was no correlation between neutralization breadth and geographical origin, age, or gender, and no correlation with reported mode of transmission or risk factor.^50^ In this same study, there was a correlation with subtype C infection and with the presence of human leukocyte antigen (HLA)‐A*03,^50^ although these two correlations were not observed in a different study.^47^ In this second study, exome sequencing performed on matched sets of individuals with and without bnAbs did not reveal any specific gene that correlated with breadth, although some candidate variants were found.^47^ To date, the data suggest that multiple factors contribute to the development of breadth in HIV‐1‐infected individuals.

### 2.3 The association between autoimmunity and HIV‐1 Infection

Early during the HIV‐1 epidemic, clinicians noted that some HIV‐1‐infected individuals would go on to develop autoimmune phenomena,^75^, ^76^ usually in the setting of uncontrolled infection. In addition, investigators noted that there was an underreporting of coincident HIV‐1 infection and systemic lupus erythematosus (SLE),^77^, ^78^, ^79^, ^80^ recognizing that lack of reporting does not mean a lack of coincident disease. It was suggested that SLE and other autoimmune diseases might provide protection against infection,^75^ but to date no study has isolated the protective factor. One candidate antibody class, antiphospholipid antibodies that can be found in autoimmune disease, was shown to block infection in vitro but testing of patient samples gave inconclusive results.^15^ Thus, whether SLE or other autoimmune diseases are protective against HIV‐1 infection remains an open question.

However, many isolated bnAbs have been shown to have autoreactivity when tested in assays used for the diagnosis of autoimmune disease^22^, ^24^, ^81^, ^82^ or when tested on arrays of human proteins.^83^, ^84^ While there are bnAbs that do not react with human antigens,^83^ most do. For some bnAbs where maturation has been studied from the initial B‐cell rearrangement to breadth, development of breadth was correlated with acquisition of autoreactivity.^24^ In one case, bnAb activity and autoreactivity were directly correlated, when a bnAb that also had reactivity with double stranded DNA was isolated from a person with coincident HIV‐1 infection and SLE,^81^ indicating that in that person, bnAb activity derived from an autoreactive B‐cell pool. In addition to autoreactivity, bnAbs usually have a high degree of somatic mutation and/or long heavy‐chain complementarity determining region 3 (HCDR3) loops,^85^ and no reported bnAb lacks all three characteristics. Autoreactivity, long HCDR3 loops, and high levels of somatic hypermutation are also associated with antibodies made by B cells that are deleted by central and peripheral tolerance controls.^86^, ^87^ Furthermore, studies of antibody knock‐in mice (engineered to have B cells with the potential to express immunoglobulins (Igs) composed of the variable region heavy (V_H_) and variable region light (V_L_) chains of mature bnAbs or their germline precursors) have demonstrated that B cells expressing many different bnAbs or bnAb precursors are under strong tolerance control,^88^, ^89^, ^90^, ^91^, ^92^, ^93^ although this does not hold for all bnAbs studied.^89^

Thus, while it may not be necessary for a HIV‐1 neutralizing antibody to exhibit characteristics of autoantibodies to have breadth, the two appear to be highly correlated. Because of this apparent correlation, we recently completed a study examining the relationship between autoantibodies and bnAb activity.^47^ In two different cohorts, we found that HIV‐1‐infected persons making bnAbs were more likely to have serum autoantibodies as well as perturbations of their circulating T‐cell populations, thus suggesting relaxed tolerance controls. T‐cell populations involved in regulation of B‐cell responses and their relationship to bnAb induction are discussed in the following sections.

## 3 T follicular helper cells and their role in bnAb induction

### 3.1 T follicular helper cells

T follicular helper (Tfh) cells are a subset of CD4^+^ T cells specialized for provision of help to B cells. Help is provided in a cognate fashion, i.e. antigen bound to B‐cell surface Ig is internalized and presented with MHC class II, and Tfh cells with specificity for the peptides presented interact with and provide help to the B cell.^94^ The first cognate interactions between B cells and CD4^+^ T cells occur at the interface between the T‐ and B‐cell zones in lymphoid tissues.^95^ T‐cell‐mediated ligation of CD40 on the B cell triggers B‐cell clonal expansion and differentiation,^96^ and cytokines produced by the T cell including interleukin (IL)‐21 and IL‐4 promote growth and differentiation and direct Ig class switching.^97^, ^98^ Provision of help is dependent on an active two‐way interaction between the B and T cell, with inducible T‐cell costimulatory (ICOS) triggering being important for IL‐21 production^99^ and IL‐4 production being dependent on signaling lymphocytic activation molecule (SLAM).^100^ Following activation, the B cells may differentiate into short‐lived extrafollicular plasma cells that have very few mutations in their Ig variable (V)‐region genes and secrete low‐affinity Ig, develop into early memory B cells, or undergo proliferation within the follicle, giving rise to GCs (reviewed in 101). GCs are comprised of B cells and Tfh cells (which migrate into GCs following initial interaction with B cells at the margin of the B‐cell follicle), together with antigen‐bearing follicular dendritic cells (FDCs), macrophages, and stromal cells. B cells undergo further cognate interactions with Tfh cells in the light zones of the GC, in which help is again mediated via two‐way receptor‐ligand interactions and production of cytokines including IL‐21.^102^, ^103^, ^104^ This enables Tfh cells to control the affinity of B cells entering the GC reaction.^105^ B cells then migrate into the dark zones where they proliferate and undergo somatic hypermutation, resulting in antibody diversification (reviewed in 106). The number of divisions B cells undergo in the dark zone and the speed of cycling are determined by the help provided by Tfh cells.^107^, ^108^ B cells then exit into the light zones, where those cells expressing surface Ig of sufficiently high affinity to enable them to efficiently acquire antigen from FDCs undergo further interactions with Tfh cells and receive signals, in particular via CD40, that enable continued survival. Tfh cells thus, select high‐affinity B cells following affinity maturation.^109^, ^110^ These B cells may then re‐enter the dark zone and undergo further round(s) of proliferation and somatic hypermutation, or alternatively become long‐lived memory B cells or differentiate into long‐lived plasma cells that typically migrate out of the lymph node (LN) to reside in the bone marrow or gut.^106^ The help provided by Tfh cells to B cells evolves over the course of the GC response: Tfh cells initially produce IL‐21 and select high‐affinity B cells, then subsequently shift to IL‐4 production and high CD154 (CD40 ligand) expression and promote B‐cell differentiation into plasma cells.^111^

### 3.2 T follicular helper cell differentiation

Tfh cell differentiation is a complex multi‐step process that is influenced by numerous heterogeneous signals (reviewed in 112). The first step occurs when naive CD4^+^ T cells are primed by dendritic cells (DCs) in LNs: an initial Tfh‐lineage fate decision is made during the first few rounds of cell division.^113^ Factors that influence CD4^+^ cell Tfh‐lineage differentiation include the cytokine environment, ICOS ligation, and signaling via the T‐cell receptor (TCR). In mice, IL‐6 plays an important role in promoting Tfh cell generation and induces upregulation of the transcription factor B‐cell lymphoma 6 (Bcl6), which is a central regulator of Tfh differentiation.^114^, ^115^, ^116^ However, although there is evidence that IL‐6 can also promote Tfh differentiation in humans,^117^ it is a not a good inducer of Bcl6 expression in human CD4^+^ T cells.^118^, ^119^ By contrast, human (but not murine) Tfh cell differentiation is potently induced by activin A^120^ and can also be driven by transforming growth factor β (TGFβ), particularly in combination with IL‐12 or IL‐23.^121^ Tfh differentiation is negatively regulated by other cytokines, including IL‐2^122^, ^123^ and IL‐7.^124^, ^125^

Tfh cell differentiation also requires ligation of ICOS by its ligand ICOSL.^126^ Studies in mice have shown that CD8α(−) DCs at the interface of the follicle and T‐cell zone play a prominent role in Tfh cell induction by upregulating expression of ICOSL and also OX40L.^127^ The G‐protein‐coupled receptor EBI‐2 positions T cells in this location, where Tfh cell differentiation is further enhanced by DC‐mediated production of soluble and membrane‐bound CD25 (the IL‐2 receptor α chain), which binds to and quenches IL‐2.^128^ Signals received via the TCR during DC‐mediated antigen presentation also influence CD4^+^ differentiation into early Tfh cells, although there is not a simple relationship between TCR signal strength and Tfh cell differentiation.^129^ Instead, the duration of signaling appears more important, with Tfh cell induction being associated with prolonged antigen presentation by DC.^130^

As CD4^+^ T cells differentiate into pre‐Tfh cells they downregulate expression of C‐C chemokine receptor type 7 (CCR7) and P‐selectin glycoprotein ligand 1 (PSGL‐1), which promote localization to and retention within the T‐cell zone, and upregulate expression of the chemokine receptor characteristic of Tfh cells, C‐X‐C chemokine receptor type 5 (CXCR5), which binds C‐X‐C motif chemokine ligand 13 (CXCL13), a chemokine produced in B‐cell follicles.^131^, ^132^ This enables pre‐Tfh cells to migrate to the margin of the B‐cell follicle,^133^ where they interact with B cells and the next step in Tfh differentiation takes place. B cells present antigen to pre‐Tfh cells, enabling further cell division. ICOSL expressed by the B cells also binds ICOS on the pre‐Tfh cell, which maintains Bcl6 upregulation, reinforcing the Tfh differentiation program and inducing directional migration of the early Tfh cells into the B‐cell follicle.^134^

As early Tfh cells undergo further interactions with B cells, GC formation occurs and they complete their differentiation into mature GC Tfh cells. Most GC Tfh cells have a CXCR5^hi^, CCR7^lo^, PSGL‐1^lo^, programmed cell death protein 1 (PD‐1)^hi^ phenotype and express high levels of Bcl6, Maf, and SLAM‐associated protein (SAP). SAP expression is essential for GC formation,^135^ preventing inhibitory signaling through SLAM family member 6 (SLAMF6) to enable Tfh‐B cell adhesion,^136^ which is crucial for Tfh cell retention in GCs and provision of help to B cells. Bcl6 plays a central role in Tfh cell differentiation,^115^, ^137^, ^138^ but other transcription factors that act upstream and downstream of Bcl6 are also required (reviewed in 139). These include lymphoid enhancer‐binding factor 1 (LEF‐1) and T‐cell‐specific transcription factor 1 (TCF‐1), which act upstream of Bcl6 to promote Tfh cell differentiation^140^, ^141^, ^142^; basic leucine zipper transcription factor ATF‐like (Batf), which positively regulates Bcl6 and is important for IL‐4 expression^143^; interferon regulatory factor 4 (IRF4), which drives differentiation of IL‐12‐stimulated CD4 T cells to Tfh rather than Th1 cells^144^; achaete‐scute homolog 2 (Ascl2), which induces CXCR5 expression^145^; c‐Maf, which enhances expression of CXCR5, IL‐21, and IL‐4^146^; signal transducer and activator of transcription (STAT)s, STAT3 and 4 being particularly important for human Tfh cell differentiation and regulation of IL‐21 expression by human CD4^+^ T cells^121^, ^147^; and forkhead box protein O1 (FOXO1), which can both promote and inhibit Tfh cell differentiation.^148^ In addition, Tfh cell differentiation is regulated by microRNAs including members of the miRNA‐17‐92 family.^149^, ^150^ Bcl6 is a transcriptional repressor and orchestrates the expression of genes involved in Tfh cell migration, differentiation, and function as well as repressing genes involved in alternative fate differentiation.^151^ Importantly, it represses B lymphocyte‐induced maturation protein 1 (Blimp 1), an antagonist of Tfh cell differentiation,^137^ as well as repressing expression of receptors for cytokines that promote CD4^+^ T‐cell differentiation into T‐helper (Th)1, Th2, Th17, and regulatory (Treg) cells. Nonetheless Tfh cells can be polarized, and subsets of Th1, Th2, or Th17‐like Tfh cells develop in certain infections/diseases.

Unlike GC B cells, which usually remain in a single GC, Tfh cells can move from one GC to another or exit the follicle altogether.^152^, ^153^ As Bcl6 expression requires ongoing induction for its maintenance, its expression is downregulated when GC Tfh cells leave the follicle, and they gradually transition to a resting memory state.^152^, ^153^ Early Tfh cells can also differentiate into memory Tfh cells without becoming GC Tfh cells. Memory Tfh cells are generated in both mice^113^, ^154^, ^155^, ^156^, ^157^ and humans^99^, ^158^, ^159^, ^160^ and can be long‐lived. They have a central memory (Tcm) phenotype and reside in spleen, lymph nodes, and bone marrow as well as recirculating in blood.

### 3.3 Circulating Tfh populations

Human peripheral blood memory Tfh cells are heterogeneous in phenotype (reviewed in 161). They are found within the CXCR5^+^ subset of circulating CD4^+^ T cells,^99^, ^159^ although CXCR5^+^ can also be expressed on non‐Tfh cells. Precise definition of circulating memory Tfh cells is challenging, as resting memory Tfh cells in blood do not express Bcl6 or the high levels of PD‐1 and ICOS characteristic of GC Tfh cells and require re‐stimulation in order to mediate B‐cell helper activity.^99^, ^159^, ^160^, ^162^ A subpopulation of blood CXCR5^+^ CD4^+^ T cells expresses PD‐1, although at lower levels than GC Tfh cells. Some of the PD‐1^+^ cells co‐express ICOS; this delineates a more activated Tfh cell population. PD‐1^+^ CXCR5^+^ CD4^+^ Tcm cells have a gene expression profile more similar to that of GC Tfh cells than PD‐1^−^ CXCR5^+^ CD4^+^ or CXCR5^−^ CD4^+^ Tcm cells and mediate superior help for B cells in in vitro culture assays.^160^, ^163^ However, memory Tfh cells are not exclusively found in this population, as PD‐1^−^ CXCR5^+^ CD4^+^ cells can upregulate PD‐1 expression and provide help for B cells if suitably re‐stimulated. CXCR5^+^ CD4^+^ Tcm cells can also be subdivided on the basis of differential chemokine receptor expression into CXCR3^+^ (Th1‐like), CXCR3^−^ CCR6^+^ (Th17‐like), and CXCR3^−^ CCR6^−^ (Th2‐like) subpopulations. The CXCR3^−^ (Th2 and Th17‐like) CXCR5^+^ CD4^+^ subpopulations provide help for B cells in vitro, whereas the CXCR3^+^ CXCR5^+^ CD4^+^ population produces little IL‐21 and has a very poor B‐cell helper capacity^99^, ^158^, ^160^, ^163^; furthermore the blood CXCR3^−^ PD1^+^ CXCR5^+^ CD4^+^ subset has a transcriptional profile most similar to that of GC Tfh cells.^160^ Nonetheless, although the CXCR3^−^ PD1^+^ subset of resting memory CXCR5^+^ CD4^+^ cells have been deemed to constitute resting memory Tfh cells,^160^ this does not provide an exclusive or comprehensive definition. Notably, CXCR3^+^ CXR5^+^ memory Tfh cells are generated under some conditions, e.g. the seasonal influenza vaccine elicits ICOS^+^ PD‐1^+^ CXCR3^+^ CXCR5^+^ Tfh cells, and the circulating frequency of this population in the periphery correlates with the quantity and avidity of the influenza‐specific antibody response.^158^, ^164^

### 3.4 Tfh cells in HIV‐1/simian immunodeficiency virus (SIV) infection

HIV‐specific CD4^+^ T‐cell responses are primed as viremia increases in acute HIV‐1 infection, but following a transient initial expansion typically decline to relatively low frequencies in the absence of antiretroviral therapy,^165^, ^166^ due in part to the preferential susceptibility of virus‐specific CD4^+^ T cells to infection by HIV.^167^ The dynamics of Tfh cell induction in acute HIV‐1 infection have not been precisely determined, but LN GCs containing proliferating Tfh cells are present in macaques by day 14 post‐SIV infection,^168^, ^169^ suggesting rapid induction of a Tfh cell response following infection.

In vitro, GC Tfh cells are more susceptible to infection with HIV‐1 than non‐GC Tfh cells or CXCR5^−^ extrafollicular CD4^+^ T cells,^170^, ^171^ and in vivo they have been shown to constitute the major CD4 T‐cell compartment for virus replication in both HIV‐1 172, 173 and SIV^174^, ^175^ infections. Furthermore, Tfh cell populations in both LNs and blood constitute a major (although not the only) site of SIV/HIV latency in macaques/humans receiving antiretroviral therapy.^171^, ^176^, ^177^, ^178^, ^179^ GC Tfh cells are not only highly activated cells that are good targets for HIV‐1 infection but are also located in close proximity to FDCs, which are an important reservoir of infectious virus and can readily transmit infection to Tfh cells.^180^, ^181^, ^182^, ^183^ Virus replication in GC Tfh cells may also be facilitated by the limited ability of antiviral CD8^+^ T cells to enter B‐cell follicles,^168^, ^171^, ^175^, ^179^, ^184^ although recent studies in the lymphocytic choriomeningitis virus (LCMV) mouse model show that persisting virus can be cleared from Tfh cells and B cells by a CXCR5^+^ CD8^+^ T‐cell population that is able to enter B‐cell follicles,^185^, ^186^, ^187^ raising the intriguing prospect that if an analogous antiviral CD8^+^ T‐cell population expressing sufficiently high levels of CXCR5 could be induced in humans, this may enable targeting of Tfh cells that harbor persistent HIV‐1 such as the latent pool.

Despite the susceptibility of Tfh cells to infection with SIV/HIV, the frequency of GC Tfh cells begins to increase within a few months of infection^188^ and elevated Tfh cell numbers are routinely observed in LNs during chronic SIV and HIV infections,^168^, ^173^, ^189^, ^190^, ^191^ although Tfh cells are eventually depleted as progression to acquired immunodeficiency syndrome (AIDS) occurs.^192^ The expansion in Tfh cells is associated with an increase in GC B cells and plasma cells and elevated IgG production, suggesting that Tfh cells contribute to the dysregulation of B cells and antibody production that occurs during HIV‐1 infection.^191^ The magnitude and duration of antigenic stimulation are known to be important determinants of the GC Tfh cell response,^193^ and although GC Tfh cell frequencies do not generally show a direct correlation with viral load, the increase in GC Tfh cells during chronic SIV/HIV infection is dependent on ongoing antigenic stimulation, as GC Tfh cell frequencies decline when viral replication is contained by antiretroviral therapy.^168^, ^189^, ^191^ Sustained LCMV replication in mice is also associated with expansion of GC Tfh cells, and here IL‐6 production has been shown to play a key role in driving Tfh cell differentiation during chronic infection.^194^, ^195^ GC Tfh cell expansion in chronic HIV‐1 infection is also associated with elevated IL‐6 production,^189^ but as IL‐6 is not such a potent stimulus of human CD4^+^ differentiation into Tfh cells, it can be speculated that upregulation of other Tfh‐differentiating cytokines, in the context of the reduction in IL‐2 expression known to occur in chronic HIV infection,^196^ may play a more important role in enhancing Tfh cell differentiation during HIV infection. Reduced control of Tfh cell numbers by regulatory T‐cell populations likely also facilitates expansion of Tfh cells during SIV/HIV infection; this is discussed further below.

Although GC responses are rapidly induced in acute HIV‐1 infection and a pronounced expansion of GC Tfh cells occurs during chronic infection, T‐cell help for B‐cell responses may nonetheless be limited. LN GC Tfh cells from chronically HIV‐infected individuals were shown to have an impaired ability to provide help for B cells in in vitro co‐culture assays, with Tfh cell helper capacity correlating negatively with viral load.^190^ GC B cells from HIV‐1 infected individuals express elevated levels of programmed cell death ligand 1 (PD‐L1), binding of which to PD‐1 on Tfh cells reduced their activation, ICOS expression, and IL‐21 production in vitro, contributing to their poor helper capacity.^190^ The functional capacity of circulating Tfh cells was also found to be impaired: the proportion of cells producing cytokines including IL‐21 was reduced, and they again exhibited a reduced B‐cell helper capacity in vitro.^163^ These defects in Tfh cell function appear to develop very rapidly, during the acute phase of HIV‐1 infection.^190^, ^197^ Analysis of both GC Tfh cells in SIV‐infected macaques^198^ and circulating CXCR3^−^ CXCR5^+^ cells in individuals chronically infected with HIV‐1^199^ suggests that Tfh cells become more Th1‐polarized by chronic infection. They also express higher levels of CD25 and exhibit enhanced IL‐2 signaling, and in vitro use of an anti‐IL‐2 antibody to block IL‐2 signaling improved the B‐cell helper capacity of circulating CXCR3^−^ CXCR5^+^ Tfh cells from infected subjects.^199^

### 3.5 Relationship between Tfh cells and bnAb induction in HIV‐1 infection

As the majority of bnAbs show evidence of extensive somatic hypermutation, it is likely that Tfh cell activity plays an important role in bnAb induction. Analysis of the relationship between Tfh cell responses and bnAb induction during HIV‐1 infection is hampered by the limited availability of longitudinal samples from individuals who eventually develop bnAbs and the difficulty in studying LN Tfh cell responses in humans. Nonetheless individuals developing bnAbs have been shown to have a higher frequency of circulating memory Tfh cells (defined as PD1^+^ CXCR3^−^ cells within CXCR5^+^ CD4^+^ T cells^47^, ^160^ or PD1^+^ CXCR5^+^ cells within CD4^+^ T cells^200^) both early (approximately 6 months) after infection and during chronic infection than individuals developing antibodies with little or no cross‐neutralizing breadth, an observation independent of the higher viral loads in subjects developing bnAbs.^47^, ^160^ Subjects developing bnAbs also have higher plasma levels of CXCL13, a biomarker of GC activity, at both early and chronic infection timepoints, than subjects who develop little/no antibody neutralization breadth.^200^, ^201^ Furthermore, in in vitro B‐cell co‐culture assays CXCR5^+^ CD4^+^ Tfh cells isolated during acute infection from subjects who subsequently developed good neutralization breadth showed induction of similar levels of antibody secretion, but higher levels of class‐switch antibodies than Tfh cells from subjects developing little/no neutralization breadth, suggesting that Tfh cell function may potentially be superior in those who make bnAbs.^200^ Although these studies only addressed total circulating Tfh frequencies and function, in macaques infected with a simian‐human immunodeficiency virus (SHIV) expressing the HIV‐1 AD8 Env (SHIV_AD8_), the frequency of Env‐specific CD154^+^ or IL‐4^+^ (but not IFNγ^+^) Tfh cells (LN PD1^+^ CXCR5^+^ CD4^+^ cells) at 44‐47 weeks post‐infection was shown to correlate positively with the concurrent frequency of IgG^+^ GC B cells and with antibody neutralization breadth at both this and later timepoints during infection.^188^ Transcriptional profiling of Env‐specific CD154^+^ Tfh cells (LN PD1^+^ CXCR5^+^ CD4^+^ cells) in SHIV_AD8_‐infected macaques also showed higher levels of Tfh‐related genes (*Bcl6*,* MAF*,* MYB*,* CXCL13* and *IL‐21*) and the Th2 gene *GATA3* (encoding GATA‐binding protein 3) and lower *Foxp3* (encoding forkhead box protein 3) in animals developing greater nAb breadth.

Together, these observations support an important role for Tfh cells in the generation of HIV‐1 antibody neutralization breadth. Nonetheless, many important questions remain about the relationship between HIV‐specific Tfh cell responses and bnAb generation. First, does the epitope specificity of Tfh cells impact on bnAb induction? As Tfh cells mediate cognate interactions with B cells, Tfh cell epitopes must be physically linked to bnAb epitopes, although they need not necessarily be in Env, e.g. in macaques primed with group‐specific antigen (Gag) plus polymerase (Pol) (Gag‐Pol) immunogens and boosted with virus‐like particles containing Gag‐Pol and Env, Gag‐Pol‐specific CD4^+^ T cells were found to enhance Env‐specific antibody production.^202^ However, as antigens can undergo degradation in vivo, it may be advantageous for Tfh cell and bnAb epitopes to be in close proximity. Second, is Tfh cell avidity important? A recent study of the influenza‐virus‐specific CD4^+^ T‐cell response indicated that T cells responding to different epitopes exhibited distinct tendencies to develop into Tfh cells, with those exhibiting a higher functional avidity being more likely to become Tfh cells^203^; but whether Tfh cells of higher avidity also mediate superior help for B cells is not clear. No associations have been reported between HLA class II type and bnAb induction during HIV‐1 infection that may support a role for T‐cell responses of particular specificity favoring or disfavoring bnAb induction^47^; but as many CD4 T‐cell epitopes are promiscuously presented by multiple HLA class II alleles,^204^ this does not preclude a relationship between epitope recognition and help for bnAb induction. Finally, how does the functional capacity of Tfh cells impact on bnAb induction? If, as discussed above, Tfh cell function is impaired in HIV‐1‐infected individuals, does this hamper bnAb generation; and/or does preservation of certain aspects of Tfh cell function favor bnAb induction during chronic infection?

Although it has not yet proved possible to elicit bnAbs by vaccination, in the RV144 phase IIb vaccine trial, priming with a recombinant canarypox vector (ALVAC‐HIV vCP1521) and boosting with a recombinant gp120 subunit vaccine (AIDSVAX B/E) were found to exhibit a 31.2% efficacy in preventing infection in a low‐risk heterosexual Thai population.^8^, ^205^ A correlates analysis revealed that IgG responses to variable regions 1 and 2 (V1‐V2) of Env associated with a decreased infection risk, while IgA responses were associated with an increased risk of infection acquisition.^206^, ^207^ Interestingly, IgG responses to V1‐V2 were higher and were associated with a decreased risk of infection acquisition only in individuals with the HLA‐DPB1*13 class II allele, while Env‐specific IgA responses were associated with an enhanced infection risk only in individuals with HLA‐DQB1*06, two class II alleles that were both common (present at frequencies of >10%) in the RV144 vaccine trial participants.^208^ Env‐specific CD4^+^ T cells directed against V2 were the most common T‐cell response induced by the RV144 vaccine regimen^209^; furthermore, RV144 vaccinees exhibited higher frequencies of circulating HIV‐specific IL‐21‐producing CD4^+^ T cells than participants in other trials of non‐protective HIV vaccines.^210^ Together, these observations suggest an important role for qualitative features of the vaccine‐induced CD4^+^ T‐cell response in determining the protective capacity of the antibody response elicited—a relationship that may prove even more critical for bnAb induction.

## 4 Regulatory cell populations and their relationship to bnAb induction

### 4.1 Regulation of GC responses

GC responses need to be precisely controlled to enable generation of high‐affinity antibodies and prevent the production of autoantibodies and development of autoimmune disease and chronic inflammation (reviewed in 211). Although the magnitude of the GC response is diminished if Tfh cell numbers are markedly reduced, limiting the number of Tfh cells is important to promote competition among B cells for interaction with Tfh cells and enable stringent selection of high‐affinity B‐cell clones. The presence of high numbers of Tfh cells results in a reduction in the selection threshold and enables survival of lower affinity and self‐reactive B cells, e.g. in *sanroque* mice homozygous for a loss of function mutation in Roquin, which results in high ICOS expression on Tfh cells and excessive Tfh cell accumulation, there is spontaneous B‐cell activation, GC formation, and autoantibody production, and the animals develop a lupus‐like phenotype.^212^, ^213^ Likewise increased numbers of circulating CXCR5^+^ CD4^+^ T cells, frequencies of which correlate with autoantibody titers, are observed in patients with SLE and other autoimmune diseases including Sjogren's syndrome and myasthenia gravis.^162^, ^214^

As discussed above, Tfh cell differentiation is a multi‐step process initiated by the signals received by naive CD4^+^ T cells as they undergo priming by DCs in LN T‐cell zones and begin to expand, and reinforced by subsequent cross‐talk with B cells at margin of and within B‐cell follicles, and within GCs. The immunologic environment at the time of CD4^+^ T‐cell priming thus has an important impact on initial Tfh induction, and regulatory cell populations including FoxP3^+^ CD4^+^ regulatory T cells (Tregs),^215^, ^216^ IL‐10‐producing regulatory B cells,^217^, ^218^ and natural killer (NK) cells^219^ may all modulate the generation of Tfh cells and hence influence GC formation and the subsequent humoral response.

However, Tfh cell help for B cells is also regulated locally within GCs. Tfh cell regulation within the GC is mediated in part by inhibitory receptors, negative feedback loops, and the availability of antigen and growth factors, and it is also regulated by specialized populations of regulatory cells. The inhibitory receptor PD‐1 is expressed at high levels on Tfh cells, and engagement of PD‐1 by PD‐L1 on B cells prevents Tfh cell proliferation following antigen recognition on B cells.^109^, ^220^, ^221^ In addition to B cells, antigen‐specific plasma cells can also present antigen to Tfh cells. However, they do not stimulate Bcl6 expression in Tfh cells and promote their differentiation into non‐Tfh cells, providing a negative feedback mechanism for reducing the GC response when large numbers of antigen‐specific plasma cells have been generated.^222^ Furthermore, as antigenic stimulation is required to sustain Tfh cell responses, the GC response is down‐modulated as antigen is cleared^193^; conversely sustained antigen persistence may promote Tfh expansion in situations of chronic infection or autoimmunity. In addition, GC responses are also regulated by regulatory cell populations that are found within the GC, including CD4^+^ T follicular regulatory cells (Tfr)^223^ and major histocompatibility complex (MHC)‐E‐restricted regulatory CD8^+^ T cells.^224^

### 4.2 Control of GC responses by regulatory CD4^+^ T cells

CD4^+^ cells expressing the transcription factor FoxP3 and high levels of CD25 play an important role in the maintenance of self‐tolerance and immune homeostasis, and the absence of FoxP3 expression results in susceptibility to development of autoimmunity, immunopathology, and lymphoproliferative disease (reviewed in 216, 225). Naive CD4^+^ T cells expressing FoxP3 are produced by the thymus. These cells, which are termed natural CD4^+^ Treg cells (nTregs) (reviewed in 225), respond to antigenic stimulation by differentiating into effector Treg cells.^226^ There are nTreg cells with specificity for both self and foreign antigens, although the ratio of antigen‐specific FoxP3^+^ to FoxP3^−^ cells is higher for the former, enabling more stringent control of responses to autoantigens.^227^ FoxP3 expression can also be induced in FoxP3^−^ naive CD4^+^ T cells in the periphery in response to antigenic stimulation in the presence of IL‐2 and TGFβ,^228^, ^229^ resulting in the generation of induced (i)Treg cells. nTreg and iTreg cells can be differentiated by expression of the transcription factor Helios, which is only expressed in nTreg cells.^230^ FoxP3^+^ CD4^+^ Treg cells can mediate control of immune responses via multiple mechanisms. They reduce CD4^+^ T‐cell activation via cytotoxic T lymphocyte‐associated protein 4 (CTLA‐4)‐mediated downregulation of CD80/86 on antigen‐presenting cells, which limits T‐cell costimulation via CD28; CD39/73‐mediated adenosine triphosphate (ATP) degradation, and CD25‐mediated IL‐2 sequestration; and can also downregulate immune responses by perforin and granzyme‐dependent lysis of antigen‐presenting cells and T cells and by production of immunosuppressive cytokines, such as IL‐10, TGFβ, and IL‐35 (reviewed in 231).

FoxP3^+^ CD4^+^ Treg cells play an important role in the regulation of humoral immune responses, as both mice and humans lacking FoxP3 have elevated levels of circulating antibodies.^232^, ^233^ By regulating initial CD4^+^ T‐cell activation and differentiation FoxP3^+^ CD4^+^ Treg cells can reduce the initial generation of Tfh cells, although they may also enhance CD4^+^ T‐cell differentiation into Tfh cells by sequestering IL‐2.^234^ However, regulatory CD4^+^ T cells are also found within GCs,^235^ and recent studies have shown that a subset of CXCR5^+^ FoxP3^+^ CD4^+^ Treg cells, termed T follicular regulatory (Tfr) cells, are generated as immune responses are induced and localize to the GC to control the GC response.^236^, ^237^, ^238^ In mice, Tfr cells have been shown to reduce antibody production and the number of GC B cells, antigen‐specific B cells, and plasma cells.^236^, ^237^, ^238^, ^239^, ^240^, ^241^ Multiple stages of the B‐cell differentiation process are inhibited, from initial B‐cell activation to the generation of class‐switched B cells and plasma cells. Tfr cells also inhibit Tfh cell expansion, differentiation, and production of cytokines such as IL‐21 and IL‐4.^239^, ^240^, ^242^ Human Tfr cells (defined as CD127^−^ CD25^+^ CXCR5^+^ CD4^+^ cells) have likewise been shown to reduce antibody production in in vitro Tfh:B cell co‐culture assays.^243^ Importantly, although Tfr cells reduce the overall magnitude of the humoral response, the antibody produced is of higher affinity than that generated in the absence of Tfr cells.^240^, ^244^

CD4^+^ Tfr cells develop from thymically derived Tregs that co‐opt a Tfh‐like differentiation pathway following activation and can also be generated from FoxP3^−^ precursors.^245^ Like Tfh cells, Tfr cell differentiation is initiated early after antigen priming by DCs. Although Tfr cells express FoxP3 and proteins characteristic of CD4^+^ Tregs including Blimp‐1, CTLA‐4, and CD39, they acquire expression of Tfh cell markers including CXCR5, Bcl6, PD‐1, and ICOS (although they do not express proteins involved in mediating help for B cells such as CD40L, IL‐21, and IL‐4).^236^, ^237^, ^238^ Their differentiation involves some of the same pathways as those used by Tfh cells, although there are differences, e.g. CXCR5 expression on FoxP3^+^ T cells is regulated by nuclear factor of activated T cells (NFAT) rather than Ascl2.^246^ Following their initial generation, Tfr cells may either leave the LN and enter the circulation to become memory Tfr cells^242^ or migrate into the B‐cell follicles where they interact with B cells and undergo full differentiation into effector Tfr cells.^239^ Tfr cell differentiation following interaction with both DCs and B cells requires co‐stimulation though CD28 and ICOS, and SAP‐dependent stimulation by B cells.^236^, ^237^, ^239^, ^247^, ^248^ CTLA‐4 expression on Tfr cells inhibits their differentiation and maintenance via interaction with CD80/86 on DCs and B cells^240^, ^249^; and PD‐1 ligation inhibits both Tfr cell differentiation and their subsequent function.^239^

It is not known whether Tfr cells regulate Tfh‐B cell interactions at the border of the follicle and modulate GC formation; but they act within the GC to suppress GC Tfh cells and B cells. The mechanisms by which Tfr cells mediate suppression of GC Tfh cells and B cells are not well defined. CTLA‐4 has been shown to play an important role in Tfr cell function,^240^, ^249^ but the importance of other receptor‐ligand interactions and suppressive mechanisms such as production of IL‐10 and IL‐35, perforin/granzyme‐dependent lysis of GC Tfh and B cells, or interference with Tfh cell interaction with B cells remain to be elucidated. Antibody responses are determined by the ratio of Tfr:Tfh cells.^239^, ^240^, ^250^ In the resting state, there is a 1:1 ratio of Tfr:Tfh cells within CXCR5^+^ CD4^+^ T cells in LNs (although lower ratios are present in the spleen and Peyer's patches). During an immune response, Tfh cells differentiate more rapidly than Tfr cells, and as GC formation occurs, Tfr:Tfh ratios decrease, typically to 1:4‐5. This decrease precedes the increase in GC B cells and antibody production. It has been speculated that as full Tfr cell differentiation is dependent on interaction with GC B cells, the increase in GC B cells may enhance Tfr cell differentiation, resulting in a feedback loop that controls the GC response.^223^ In autoimmune BXD2 mice, high levels of IL‐21 enhance Tfh cell generation and drive a decline in the Tfr:Tfh cell ratio, promoting autoantibody production.^251^

### 4.3 Control of GC responses by regulatory CD8^+^ T cells

Studies in mice have identified a subset of CXCR5^+^ CD8^+^ T cells in LN GCs that also contribute to the regulation of humoral responses (reviewed in 224). These cells have been shown to suppress T‐cell‐dependent B‐cell responses in a H‐2 Qa‐1‐dependent manner and play an important role in maintenance of self‐tolerance.^252^ Qa‐1 and its human homolog HLA‐E are non‐classical MHC class Ib molecules that predominantly present a peptide derived from the signal sequence of classical MHC class Ia molecules, recognition of which by the inhibitory NK cell receptor NKG2A/CD94 contributes to inhibition of triggering of NK cell effector activity on contact with cells that express normal levels of MHC 1a molecules.^253^ However, MHC‐E can also present some peptides derived from autoantigens and pathogens, which are recognized by CD8^+^ T cells. Qa‐1 is expressed at high levels on GC CXCR5^+^ CD4^+^ cells, while CXCR5^−^ CD4^+^ T cells express very low levels of Qa‐1; hence, Qa‐1‐restricted CXCR5^+^ CD8^+^ T cells specifically target CXCR5^+^ Tfh cells.^254^ By mediating perforin‐dependent lysis of GC Tfh cells, they reduce the GC response and help to prevent autoantibody development.^252^, ^254^, ^255^ Although Qa‐1‐restricted CD8^+^ Treg cells express CXCR5, they do not express ICOS or PD‐1 or markers characteristic of CD4^+^ Treg cells such as FoxP3. However, they do express ICOSL and CD122 (the IL‐2 receptor β chain, which also forms part of the IL‐15 receptor).^224^ The mechanisms involved in their differentiation are not fully understood, although Helios‐dependent STAT5 activation has been shown to be important to enable their survival and prevent terminal differentiation.^256^ Whether similar CXCR5^+^ CD8^+^ Treg cells contribute to regulation of GC responses in humans remains unclear, although HLA‐E‐restricted CD8^+^ Treg cells have been suggested to be involved in the control of type 1 diabetes in humans.^257^

### 4.4 CD4^+^ Treg cells and CD4^+^ Tfr cells in HIV‐1/SIV infection

FoxP3^+^ CD4^+^ Treg cells have potential to mediate both beneficial and detrimental effects during HIV‐1 infection, by suppressing the generalized T‐cell activation and inflammation that contributes to ongoing virus replication and disease progression and/or impairing HIV‐specific immune responses. However, despite considerable investigation, their roles remain incompletely understood (reviewed in 258). Analysis of regulatory CD4^+^ T cells in HIV‐1 and SIV infections is complicated by the difficulty in distinguishing CD25^+^ FoxP3^+^ CD4^+^ Treg cells from activated conventional CD4^+^ T cells, as the latter also express CD25 and FoxP3 transiently following activation.^259^

Despite the fact that CD4^+^ Treg cells can be infected with HIV‐1 and the absolute number of both total CD4^+^ T cells and CD4^+^ Treg cells is reduced in HIV‐1‐infected individuals, multiple reports indicate that the frequency of CD4^+^ Treg cells within circulating CD4^+^ T cells is increased.^260^, ^261^, ^262^, ^263^, ^264^, ^265^, ^266^, ^267^, ^268^ Enhanced generation of nTreg cells from the thymus,^269^ increased iTreg induction in the periphery,^270^, ^271^ and enhanced CD4^+^ Treg cell survival^272^ have all been suggested to contribute to this increase. Furthermore, as HIV‐specific Tregs are induced during infection,^273^, ^274^ the increase in CD4^+^ Treg frequencies may be, in part, driven by antigenic stimulation. CD4^+^ Treg cells have also been found to accumulate in tissues during HIV‐1 infection.

The in vitro suppressive capacity of CD4^+^ Treg cells from HIV‐1 infected individuals is reported to be comparable to that of HIV‐seronegative donors, and CD4^+^ Treg cells from subjects acutely or chronically infected with HIV‐1 have also been shown to suppress HIV‐specific CD4^+^ T‐cell proliferation and cytokine production in vitro.^274^, ^275^ However, the circulating frequency of CD4^+^ Treg cells has not been found to show any correlation with the magnitude of HIV‐specific T‐cell responses,^276^, ^277^, ^278^ leaving the contribution of CD4^+^ Treg cells to regulation of virus‐specific T‐cell responses in HIV‐infected individuals unclear. Their role in suppressing immune activation is also uncertain. Many studies report a positive correlation between CD4^+^ Treg cell frequencies and levels of T‐cell activation^260^, ^262^, ^263^, ^279^, ^280^, ^281^, and CD4^+^ Treg cell frequencies also correlated inversely with the circulating CD4 T‐cell count. However, this may reflect enhanced CD4^+^ Treg generation in the context of immune activation (and potentially also enhanced virus replication, although CD4^+^ Treg cell frequencies are not always reported to correlate with plasma viral load).

Relatively few studies have addressed Tfr cell frequencies during HIV‐1 or SIV infection. The frequency of Tfr cells within total LN CD4^+^ T cells does not appear to be increased during chronic SIV^282^, ^283^ or HIV‐1 infection,^284^ although the absolute number of Tfr cells is increased. One report suggests that the Tfr cell frequency in the spleen may be increased during HIV‐1 infection, however.^285^ Two studies in which the LN Tfr:Tfh cell ratio was analyzed in macaques chronically infected with SIV report conflicting results, with one finding an increase^284^ and the other a decrease.^283^ In the latter study, the decrease in the Tfr:Tfh cell ratio correlated with an increase in the percentage of GC B cells, suggesting that Tfr cells suppress GC B‐cell formation. LN Tfr:Tfh cell ratios have not been investigated during HIV‐1 infection, but the ratio of Tfr:Tfh (i.e. CD25^+^ FoxP3^+^:CD25^−^ FoxP3^−^) cells within the circulating CXCR5^+^ CD4^+^ T‐cell pool in a cohort of chronically infected individuals was found to be similar to that of uninfected subjects.^47^ Whether there are alterations in Tfr cell function during HIV‐1/SIV infections remains to be investigated; however, in experiments in which tonsil cells were infected with HIV‐1 in vitro, Tfr cells were found to express increased levels of CTLA‐4, lymphocyte activation gene 3 (LAG‐3), IL‐10, and TGF‐β and retained the capacity to impair Tfh cell proliferation and production of IL‐21 and IL‐4.^284^

### 4.5 Relationship between CD4^+^ Treg cells/CD4^+^ Tfr cells and bnAb induction in HIV‐1/SIV infection

Regulatory CD4^+^ T‐cell populations, in particular Tfr cells, control the overall magnitude of the GC response and regulate the stringency of B‐cell selection within GCs, limiting the production of lower‐affinity antibodies and auto‐reactive antibodies. They may, therefore, constrain the production of HIV‐1 bnAbs, which, as discussed earlier, commonly exhibit extensive somatic hypermutation and show evidence of autoreactivity, suggesting a need for strong GC Tfh cell activity and relaxed selection controls for their generation. One study in SIV‐infected macaques found that circulating CD4^+^ Treg numbers correlated positively with autoantibody titers and the spectrum of autoantigens recognized, which does not support a relationship between relaxation of host tolerance controls and generation of antibodies with greater autoreactivity^286^; however, LN Tfr cell numbers and the Tfr:Tfh cell ratio were not addressed. Conversely in another SIV study, the LN Tfr cell frequency (% FoxP3^+^ CD25^+^ CXCR5^+^ CD4^+^ T cells) was found to show an inverse correlation with the frequency of LN Tfh cells and the avidity of antibodies recognizing the SIV gp120 envelope protein in plasma, indicating a role for Tfr cells in constraining the maturation of the envelope‐reactive antibody response.^282^

We recently analyzed circulating CD4^+^ Treg and CD4^+^ Tfr cell populations in subjects chronically infected with HIV‐1 who had generated bnAbs and matched individuals who had developed little or no antibody neutralization breadth.^47^ The frequency of CD4^+^ Treg cells within lymphocytes was significantly lower in the bnAb group, although there was no difference between groups in the percentage of CD4^+^ Treg cells within CD4^+^ T cells, the circulating frequency of Tfr cells, or the Tfr:Tfh ratio (ratio of CD25^+^ FoxP3^+^:CD25^−^ FoxP3^−^ cells within circulating CXCR5^+^ CD4^+^ T cells). Interestingly, however, PD‐1 was expressed at significantly higher levels on both CD4^+^ Treg and CD4^+^ Tfr cells in the subjects who had generated bnAbs. As PD‐1 inhibits the function of CD4^+^ Treg and CD4^+^ Tfr cells,^239^ this suggested that the suppressive capacity of CD4^+^ Treg/Tfr cells in the bnAb‐producing subjects may be impaired, an observation supported by the finding that the PD‐1^hi^ subset of CD4^+^ Treg cells from some HIV‐seronegative donors exhibited an impaired capacity to inhibit the proliferation of conventional CD4^+^ T cells in vitro.^47^ As discussed above, autoantibodies were also detected in a higher proportion of the group of subjects who produced bnAbs than those who did not. Together, these findings suggest that bnAb development during HIV‐1 infection may be favored by a relaxation in regulatory CD4^+^ T‐cell control of antibody production that enables a strong GC response and permits some degree of autoreactive antibody production.^47^

## 5 Implications for design of bnAb‐inducing vaccines

**Figure 1:**
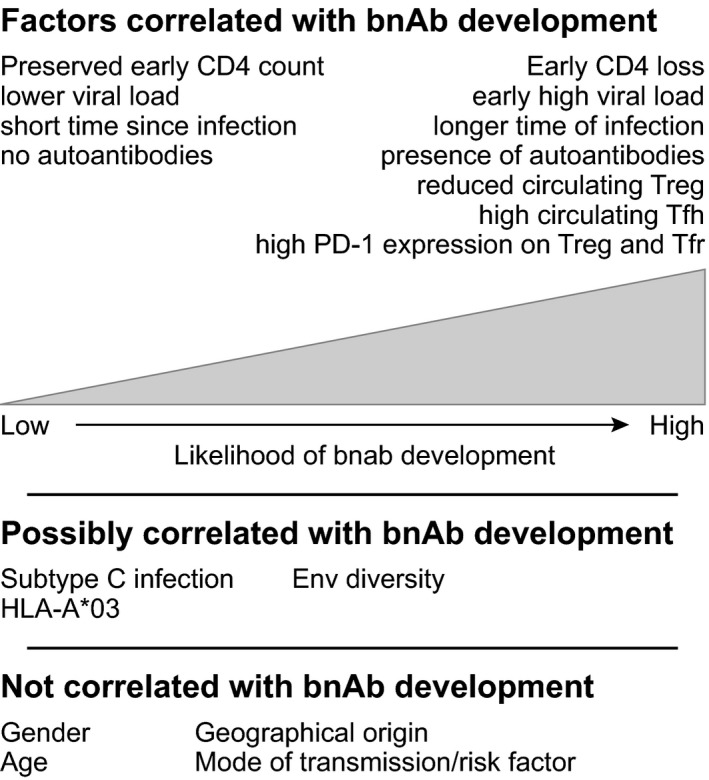
Factors associated with bnAb development in HIV‐1‐infected individuals. HIV‐1‐infected individuals who make bnAbs have been found to have perturbations of their immune system. In contrast, demographic characteristics have not been found to correlate with bnAb development

A major focus of efforts to develop bnAb‐inducing vaccines has been on antigen design.^287^, ^288^, ^289^ Dissection of the pathways by which bnAbs evolve in HIV‐1‐infected individuals has led to recognition of the need for antigens that are able to trigger B cells expressing the unmutated common ancestors of mature bnAbs, drive the selection of daughter cells expressing antibodies with potential for further maturation along bnAb lineages, and ultimately enable bnAb evolution.^34^ However, in addition to design of appropriate vaccine antigens, immunization strategies need to be devised that will elicit an environment supportive of an appropriate B‐cell response to them. Here too, insights can be gained from analysis of HIV‐1 infected individuals who develop bnAbs. Factors shown to correlate with bnAb induction in HIV‐1 infection are summarized in Figure 1. Together they suggest that a strong CD4^+^ Tfh cell response, reduction in host tolerance controls, potentially mediated by a reduction in regulatory CD4^+^ T‐cell suppression of GC responses, and strong, sustained antigenic stimulation are all of importance to enable a potent GC response and drive B cells to undergo multiple rounds of somatic hypermutation, in the context of a relaxation of host constraints on autoreactive antibody production.

Vaccine development efforts are, thus, likely to benefit from consideration of these three inter‐related factors. First, a strong antigen‐specific CD4^+^ Tfh cell response needs to be elicited. As discussed above, it is currently unclear whether this should ideally target particular CD4^+^ epitopes within the vaccine antigen, although it can be speculated that targeting of high‐affinity epitopes in close proximity to bnAb epitopes may be beneficial. However, the use of immunization regimes (e.g. vectors and adjuvants) that promotes CD4^+^ T‐cell differentiation into Tfh cells will be of great importance. For example, recent studies in murine models demonstrated that adenoviral vectors elicited a strong Tfh cell response to encoded vaccine antigens,^290^ and that adenoviral priming enabled induction of strong humoral responses to a subsequent protein boost delivered with a relatively weak adjuvant^291^; and adenoviral vectors have also been shown to elicit strong Tfh cell responses to encoded HIV antigens in macaques.^292^ Second, immunization platforms need to be developed that will enable high level and prolonged antigen availability. This is of importance to sustain the Tfh cell response and the GC reaction and enable B cells to undergo multiple rounds of somatic hypermutation. Repeat immunizations, vectors that drive prolonged antigen expression, and/or platforms enabling slow antigen release over time (e.g. nanoparticle delivery systems^293^) may all be helpful. Third, it may be necessary to transiently modulate host constraints on autoreactive antibody development at the time of bnAb induction to enable the evolution of bnAbs whose development is constrained by host tolerance controls. For example, this could be achieved by the use of immune modulatory strategies such as transient CTLA‐4 blockade,^294^ although this type of approach will need to be carefully safety‐tested in animal models prior to study in human trials.

In summary, study of the mechanisms that enable bnAb induction in some HIV‐infected individuals has given important insight into how bnAb induction may be achieved by vaccination. The benefit to rational vaccine design strategies is such that achievement of bnAb induction by vaccination now seems very likely.

## Conflicts of interest

The authors have no conflicts of interest.
